# Altruistic behaviors and cooperation among gifted adolescents

**DOI:** 10.3389/fpsyg.2022.945766

**Published:** 2022-08-11

**Authors:** Ashraf Atta M. S. Salem, Mahfouz Abdelsattar, Mosaad Abu Al-Diyar, Amthal H. Al-Hwailah, Esraa Derar, Nadiah A. H. Al-Hamdan, Shouket Ahmad Tilwani

**Affiliations:** ^1^College of Management Sciences, Sadat Academy for Management Sciences, Cairo, Egypt; ^2^Hurghada Faculty of Education, South Valley University, Qena, Egypt; ^3^Department of Psychology, Suez University, Suez, Egypt; ^4^College of Social Sciences, Kuwait University, Kuwait City, Kuwait; ^5^Department of English, College of Science and Humanities, Prince Sattam Bin Abdulaziz University, Al-Kharj, Saudi Arabia

**Keywords:** cooperation, cooperation theory, indirect reciprocity, social desirability, altruistic behaviors, kin selection, reciprocal altruism, giftedness

## Abstract

The present study is a differential study that describes the nature of the relationship between cooperation and altruistic behavior in a sample of gifted adolescents in three universities in Egypt and Kuwait University. It also identified the differences between males/females, and senior students/junior students in both cooperation and altruism. A total of 237 gifted adolescents—with average age 21.3 ± SD 2.6 years—from three Egyptian universities: Alexandria University, Sadat Academy for Management Sciences, and Suez University (in Egypt), and Kuwait University, were involved in this study. Measures used in the study include the Scales for Rating the Behavioral Characteristics of Superior Students (SRBCSS), Generative Altruism Scale (GAlS), and The Cooperative/Competitive Strategy Scale (CCSS). Results revealed that there is a significant positive relationship between altruism and cooperation among gifted adolescents. Also, findings show that there are statistically significant differences between males and females in both altruism and cooperation. In addition, there are differences statistically significant between senior students and junior students in both altruism and cooperation in favor of senior students. It is recommended that altruism and cooperation intervention-based programs should be designed to increase the adaptive behaviors of adolescents.

## Introduction

Regardless of the diversity of giftedness theories, gifted teachers generally consider giftedness to be abnormally high ability within any area, which includes high global IQ and domain-specific capability. They tend to strive to become inclusive in determining gifted and skilled children and youngsters by adopting obviously defined but wide conceptions of giftedness and the use of several alternative requirements along with assessment over period, given the several manifestations of giftedness. It is also recognized that giftedness can manifest itself in various ways in various ethnic or socioeconomic groups, as well as in various cultural settings. Researchers and professionals have called for a shift in primary of talented education to skill development, partly due to the diversity in ideas of giftedness and identification procedures, and partly due to the collateral significant informative provisions. Because of this, skill development emphasizes the development of all students' talents, items, and capabilities, including highly able or high-end learners (Chan, 2015).

Skilled and talented students are those who have been discovered by professionals as having exceptional talents and are ready of powerful. Those children who, to realize their contribution to themselves and their society, require differentiated educational programs and/or services different from those provided by the regular school program. Folks who demonstrate many of the following talents or aptitudes in any of the pursuing areas, singly or in combination, can handle high performance: (1) general intellectual potential, (2) specific academics aptitude, (3) creative or productive pondering, (4) leadership potential, (5) visual and performing arts characteristics, and (6) psychomotor ability (Daniels and McCollin, 2010, p. 2).

Besides focusing on the intellectual aspects of giftedness, the tradition of studying the social aspects of giftedness has given rise to research on social giftedness (Renzulli, 1986). When it comes to the development factors of creative potential and research thinking, J.S. Renzulli emphasizes the importance of the social aspects of giftedness (Borodina and Solomatin, 2015). There are several social manifestations of giftedness such as cooperation and communication as being associated with curiosity, preferred learning styles and self-expression, self-regulation, planning, and learning pleasure (Kirby et al., 2011).

Individuals' social giftedness and other types of giftedness develop over time, resulting in their social significance and the possibility of using their talents to better the world (Konrath et al., 2010). The psychology of social giftedness is concerned with a personality's exceptional ability to form mature, constructive relationships with others (Gudzovskaya and Shpuntova, 2016). In accordance with Bogoyavlenskaya et al. (2003), giftedness manifests itself in a sedentary lifestyle, based on the systematic approach, therefore, in all public groups and spheres (family, business contact in politics, business relations in collectives), social giftedness is quite vividly put in communicative, command, and spiritual-value activities (Courtinat-Camps et al., 2017). Gifted individuals are frequently related to different psychological personalities and emotional issues. They were also related to social issues in their classroom, such as adjustment, remote location, and acceptance by peers (Yoo and Moon, 2006; Ishak and Abu-Bakar, 2010). Indeed, talented students are thought to wrestle with some humanistic skills, such as empathy, which can make hard for them to form positive social romantic relationships with the peers.

Based on this social perspective of giftedness, gifted is supposed to show high levels of prosocial behaviors, mainly cooperation and altruism that may help as a psychological buffer and act as prevention or immunity in face of stressful events in their daily life and interactions with other people (Guenther, 2006; Gagné, 2009). Prosocial behavior is a voluntary, intentional behavior that benefits others, it is the “social glue” that allows people of all ages to live peacefully and productively together (Eisenberg and Miller, 1987, p. 92). Prosocial behavior can take many forms, from small acts of kindness, like letting someone in a hurry go ahead at the cashier, to longer-term acts, like volunteering for a charity, and even everyday tasks, like taking care of one's grandchildren. But, as the previous example shows, the reasons why people do good things can change over time (Lay and Hoppmann, 2015).

Prosocial behavior encompasses a wide range of behaviors aimed at benefiting one or more people other than oneself, such as assisting, comforting, sharing, and cooperating. Altruism is motivated by a desire to improve the welfare of others, as opposed to egoism, which is motivated by a desire to improve one's welfare. Prosocial behavior and altruism do not have a one-to-one relationship. Prosocial behavior does not have to be motivated by altruism, and prosocial behavior does not have to be motivated by altruism. Altruistic behavior is viewed as that which is motivated by a genuine desire to help another person without expecting anything in return (Feigin et al., 2014).

As an interpersonal construct, altruism is linked to prosocial behavior. While altruism is defined differently by each discipline, it is often defined as an action taken to help another. Altruism refers to a motivational state that aims to improve the welfare of others, egoism, or the desire to improve one's welfare, opposes altruism. An increasing body of research has focused on why humans engage in prosocial behaviors such as altruism when it is often counter to their self-interest and sometimes their wellbeing. A lot of people think of altruism as a uniquely human trait that is closely related to the tendency to cooperate with other people.

Altruism is often connected to ideas like punishment, reward, reciprocity, and working together. Cooperation depends on altruistic punishment, which is a powerful social tool that can get social outcasts to act like good citizens. Altruism and related ideas like cooperation and reciprocity are often seen as traits that only humans have. Altruistic punishment is when someone breaks a social rule and is punished for it, usually by a third party or someone who is not directly affected by the violation (Van Dyne and LePine, 1998; Fehr and Gächter, 2002).

Therefore, altruism and cooperation among unrelated individuals are of paramount importance due to their widespread occurrence, it is based on the principles of natural selection. Eishenberg et al. (1999) argued that altruism and cooperation are two clear manifestations of prosocial behaviors, that contribute to preserving the survival and development of the society. Accordingly, the present study investigates the cooperation and its relationship with altruism among a sample of gifted students in four different universities: Alexandria University, Sadat Academy for Management Sciences, and Suez University (in Egypt) and Kuwait University (State of Kuwait).

### Significance of the study

Gifted students contribute significantly to the advancement of human culture and civilization. Gifted and talented are in high demand, they are in the spotlight from an early age, and they can go on to achieve great things in any field they choose. One in four young people born with high intelligence are not able to fully realize their potentials because of inadequate supporting environment (Chalshtari and Heidari, 2016). The society around them may be unaware of their social and emotional characteristics. Research shows that some gifted students feel insecure and anxious when they don't have the right environment. This can also lead to retardation, poor concentration, isolation, aggression, and even a lack of activity and passivity (Ahmadi et al., 2012). Gifted and talented usually feel happy and content with their lives because of their social and psychological traits, such as helping others, being patient and persistent, being sensitive, and being interested in works of art.

There are numerous conceptualizations of giftedness including conceptions of wellbeing (e.g., life satisfaction, happiness, and quality of life), that supports prosocial behaviors among University students. Social giftedness includes the capacity to form mature, constructive relationships with other individuals and groups, exemplified by consistent prosocial behavior (Bergold et al., 2015; Gudzovskaya and Myshkina, 2022). Therefore, a differential study identifying the difference between male and female, junior and senior gifted University students is crucial especially in the Arab region and Middle East.

## Literature review

### Altruistic behaviors

Behavior is positive when it is desirable and beneficial to others. Positive psychology has grown in popularity in recent years, and researchers have become increasingly interested in developing a thorough understanding of positive behavior. Attention has been paid to personality-related behaviors, such as helping behavior and other behaviors that facilitate coexistence with others and foster positive social relationships, such as altruistic behavior and empathy (Zheng et al., 2016, p. 1,575).

Altruistic behavior is one of the most important aspects of positive personality development. Research has been conducted on the relationship between altruism and social responsibility (Pavenkova et al., 2015; Kim and Han, 2018). Altruistic behavior entails empathizing with and helping others without expecting anything in return, out of a sense of social responsibility toward them. Social responsibility is a value that motivates individuals to engage in positive social and moral behaviors, relationships with others, and the application of care and justice principles, allowing the individual to strike a balance between empathy for others and concern for justice (Wary-Lake and Syvertsen, 2011, p. 12).

#### The evolution of altruism

Typically, the situation of altruism or self-neglect has been scrutinized in biology since 1872, when Charles Darwin used his theory of progress, based upon natural and sexual selection, to make clear what he termed “moral sense.” In his own words, “any animal endowed with well-marked social nuggets of information, parental and filial affections included, would inevitably get a moral sense, as soon as its perceptive power became as developed, or as near developed, just as man” (Darwin, 2002, p. 121).

Since then, biologists have investigated this topic from the standpoints of development, physiology, and genes, among other natural sciences. During the nineteenth and twentieth centuries, this viewpoint gave rise to several theories to explain altruism, including parental selection and reciprocal altruism. Nonetheless, researchers in major psychology and behavior genetics are still attempting to shed light on altruism, and a substantial amount of knowledge has been produced in the process of comprehending our altruistic behavior (Perez Bernardes de Moraes and Dos Santos Millani, 2014).

The development of behavioral patterns that include altruistic decisions which are referred to as “moral sense” provides them with some adaptive advantages within their social environment, and occasionally within other groups of people. It is still common for social scientists to express reservations about the spread of moral sense and cultures (Jablonka and Lamb, 2005, 2006, 2007, 2008). Social teamwork was critical for early hominid adaptation during hunts, wars, and environment exploration. This kind of behavior kept people from fighting and made it easier for them to share food. To keep a group together, people stopped relying on a sense of reciprocity and started punishing partners in different social contracts. In this situation, humans' brains have changed to help them process social information. People's minds are set up to solve the problems that our ancestors had to deal with. Our minds have been shaped by hundreds of thousands of years of environmental pressure through natural and sexual selection (Tooby and Cosmides, 1997; Miller et al., 2000; Kanazawa, 2008).

Accordingly, altruism is a social construct that is associated with numerous forms of prosocial behavior. Altruistic behavior, such as assisting a stranger in need, is regarded as a critical component of cooperation in human societies. However, our propensity for altruistic acts varies significantly between individuals, ranging from extremely altruistic kidney donors to extremely asocial psychopaths. Altruism is typically opposed to egoism, which is typically motivated by the desire to maximize one's own health. Understanding why humans engage in prosocial behaviors such as altruism even though they frequently contradict our self-interest and occasionally our wellbeing is a growing area of behavioral and neurological study.

#### Concept of altruism

Humans frequently show altruism toward strangers who are unlikely to return the favor. Many people, often anonymously, donate blood and money to help people they have never met. People frequently cooperate with strangers in one-shot prisoner's dilemmas (where “defecting” always yields a higher individual payoff) and offer something rather than nothing to strangers in dictator games in experiments (when they could have kept everything for themselves; Camerer and Thaler, 1995; Henrich et al., 2001; Camerer, 2003; Fehr and Rockenbach, 2004; Gächter and Herrmann, 2008). There are a lot of people who are willing to make sacrifices in order to exact revenge on those who have caused problems for the community or for other people. Another type of altruism is demonstrated here (Fehr and Gächter, 2002). Even though altruistic behaviors can look very different from one society to another, being kind to others is a characteristic shared by all people everywhere (Gächter and Herrmann, 2008; Vakoch, 2013).

While the definition of altruism varies by discipline, it is frequently defined as an action taken to assist another. In essence, biologists and evolutionary scientists are frequently concerned with the utility of a particular behavior, whereas psychologists are concerned with the motivation for the behavior (Isúmen and Yldz, 2005; Filkowski et al., 2016). From an evolutionary point of view, altruism is a behavior that lowers the fitness of an individual or their genetic contribution while simultaneously raising the fitness of another (De Waal, 2008). According to studies conducted in the field of psychology, altruism is defined as a motivational state in which a person acts with the intention of improving the welfare of another person (Wilson, 1992).

Altruism is defined as both a self-sacrificed connection to another person and a self-sacrificed act to assist another person. Altruism is also “a state of love directed toward others instead of egoism and self-indulgence” (Hançerlioglu, 1978; Enç, 1990). The primary criterion for defining the term “altruism” is the presence of a desire to assist (Onatir, 2008). Intention to assist and accountability are critical characteristics of a person who tries and ultimately satisfies himself/herself for the benefit of disabled persons. Thus, altruism is a social behavior system founded on the moral values of mercy, humility, and a desire to assist others. The central concept of altruistic behavior is that altruistic behaviors entail charitable acts, i.e., making good deals for the sake of goodness (Pavenkova et al., 2015).

One of the most important parts of altruism is love for other people. Sorokin (1967) idea of altruism envisages that there are six kinds of love: (1) Religious love is feeling God's love; (2) Ontological love is using love to unite, harmonize, elevate, enrich, and empower; (3) Ethical love is identifying love with values like goodness, truth, and beauty; (4) Biological love is love expressed sexually through passions; (5) Psychological love is love experienced emotionally through arousal; and (6) Spiritual love is love experienced spiritually.

#### Types of altruism

Not only in humans, altruism can be traced in most animals, but it is usually limited to family members. Some species, however, go further than that. On the other hand, humans do this with most of their own kind. It's important to remember that the way people divide up their work creates complex relationships. At that time, people seemed to have specialized roles and had already divided up the work. During a hunt, for example, one person might have been a master at making arrows, while another might have been a master at spear throwing, and yet another might have been a master at planning (Ridley, 2000, p. 50–61).

Altruism and moral behavior in the context of other animals has enriched our perception of such phenomena, as we can observe similarities between our and their moral behavior, especially regarding the three pillars upon which altruistic behavior is founded in the animal kingdom, including human altruism. These three pillars are as follows: (1) selection of kin, (2) mutual altruism, and (3) fitness metric (Perez Bernardes de Moraes and Dos Santos Millani, 2014).

##### Kin selection

The main point of this theory is that we tend to like people who are related to us. Modern biology says that this is because we share a larger part of our genome with them. This means that favoring them would be favoring our traits. The theory of kin selection was first put forward by W. D. Hamilton in 1964. The term “kin selection” suggests that Darwinian selection may affect not only individuals but also families, but what it really shows is that natural selection happens at the gene level, so when we favor our family, we are favoring the same things we favor when we act selfishly, which are our genes (Wright, 2010).

Thus, from a genetic perspective, assisting copies of our genes located in another person's body produces the same results as assisting these genes in our own bodies (Okasha, 2006; Wright and Jones, 2006; Wright, 2010). According to Dawkins, genes that can aid in the propagation of their copies in other bodies give the appearance of altruism, but it is a genetic manifestation of selfishness (Dawkins, 2001, p. 113). However, theorists have identified one set of circumstances in which cultural kinship can promote cooperation: in groups dominated by a single highly prestigious individual and in which individuals look to this individual for behavioral cues, cooperative actions can become and persist (Henrich et al., 2015; Henrich, 2016; Gächter and Renner, 2018).

As a result, by helping our family, we are helping the people who have the greatest chance of having our genes in their bodies, and this is a genetic benefit of altruism. Remember that biological functions don't necessarily translate into biological motivations. For example, a person who exhibits sex drive toward someone of a different gender isn't motivated by the desire to perpetuate his or her genes; rather, sex drive is a motivating factor. The same holds when someone experiences the sensation of sex with another person. Altruism in human societies is not limited to this type of altruism; kin selection can explain some, but not all, of it. Human societies have relied on the social exchange since the dawn of time to ensure that individuals have access to resources that allow them to live healthier and longer lives.

Social interaction is so fundamental to the evolution of our species that in the earliest stages of our species' evolution, natural selection developed neural and cognitive mechanisms to facilitate it. According to this theory, humans have specialized cognitive mechanisms for detecting cheating in social exchanges, making us more likely to seek out trustworthy and cooperative partners rather than cheaters (Stanovich and West, 2004; Barbey and Barsalou, 2009; Jaaskelainen et al., 2011).

##### Reciprocal altruism

Robert Trivers, a biologist, observed in the late 1960's that animals, like humans, could benefit from cooperation if they interacted long enough to develop the necessary trust. They would be much better off in the long run if they shared their resources with those who do the same and fulfilled their contract than if they cheated and gained immediate advantage. Trivers argued that repeated interaction strengthened the behavior which is called reciprocal altruism (Trivers, 1971).

Therefore, reciprocity refers to actions that may appear harmful to oneself but beneficial to another, with the expectation that the other party will act similarly in a subsequent interaction. In small, isolated groups where repeated interactions are possible, reciprocal altruism is more likely to manifest. A strong reciprocator adheres to a group's social norms and, consequently, punishes partners or group members who violate social norms. Even when there is no obvious benefit to cooperating, strong reciprocators are inclined to do so. There is evidence that rewarding and punishing others based on social norms leads to group cooperation. The effects of punishment can be observed in future interactions where individuals who have previously been punished increase cooperation with new partners (Fehr et al., 2002).

Reciprocal altruism exists not only between members of the same species, but also between partners from different species. Mutualism or cooperation is a term used in ecology to describe a relationship between individuals of different species in which both benefit (reciprocal altruism; Ridley, 2000, p. 72–79). However, human altruism extends beyond kin selection and reciprocal altruism to include kindness to people who are not genetically related to us and who will never be able to repay us, and similar behavior can be found in other animal species.

##### Altruism as a fitness indicator

“Fitness indicators are signals of an individual's characteristics and quality that can be perceived by others” (Miller, 2012, p. 24). Fitness indication through altruistic behavior can be paid as social status in any social group, such as human society. Within groups of humans, observers can always see hierarchy developing, particularly in the division of labor between males and females. Humans have a more egalitarian hierarchical stratification than other primates, especially when compared to chimps, our closest relatives (Wright, 1997; De Waal et al., 2000; de Waal, 2001; Fuentes, 2006; Perez Bernardes de Moraes and Dos Santos Millani, 2014). Altruistic behavior can be extremely beneficial in this context of defined hierarchy, not only reciprocally, but also by demonstrating to the group the abilities that an individual possesses that may be of group interest, resulting in increased social status, sex, resources, and other conceivable social benefits for that individual.

The increase in social status is another advantage of investing in altruistic behavior as a fitness indicator (Smith and Winterhalder, 1992). Individuals whose altruistic behavior has become the defining mark of their personality, allowing them to achieve enormous social status within their social group, can be found in all known societies past and present (Gintis, 2000; Anderson et al., 2001; Caravita et al., 2009). It's worth noting that human survival and reproductive success are inextricably linked to social status, emphasizing the importance of investing in altruistic behavior toward any member of a social group (Miller et al., 2000; Harris, 2002).

Altruism is a preferred source that aims at preferring the good of others to the personal good; it is the direct opposite of selfishness. This means that an individual's interest and love tendencies are directed toward the others before themselves, whether it was primitiveness or acquisition (Omar, 2008, p. 60). The motive for altruism is influenced by social and cultural factors as well as personal factors since the individuals prefer others over themselves to make the world a better place to live in. Altruistic individuals laugh for others and give up time, money, and place, motivated by faith in people and support for their independence. For the sake of their human perspective, they give up internal obstacles and the ones imposed on them by others and work to live a life in which their internal values are along with their external decisions and work, which creates in them a motivation to achieve their altruistic vision (Tyink, 2006, p. 6).

By analyzing the effect of a short intervention on 6- to 7-year-old children, the plasticity of altruistic behavior in children was investigated (Lozada et al., 2014). Beyond reciprocity and reputation, the intervention positively influenced children's altruistic behavior. Collaboration, emotional security, and moments of relaxation all increased participants' awareness of themselves and others, which favored the emergence of intrinsic altruism. This is consistent with our theory that altruism is an embodied human resource that is highly susceptible to social context. In gifted adolescents, on the other hand, factors that influence acts of altruism in a sample of gifted female adolescents in Singapore include personality factors and value system (empathy, high sense of justice, and optimism), social skills, and social factors (family, school culture, and service-learning experiences; Pramathevan and Bacsal, 2012).

### Cooperation behaviors

Cooperation is viewed as a prosocial behavior that is manifested in human interactions among people in society. Cooperation is considered among the social operations important to the stability of social life and peacefully establishes relations between the individuals and responsible and working together to achieve public purposes. Cooperation differs in terms of scope since it could be limited to a group of individuals or a factory or a local environment, and its scope could be extended to include a province or several countries. The strength of a group is measured through the presence of safe foundations and accurate systems, it is consolidated through cooperation and solidarity among its members to achieve public interests and common good since the non-cooperation is the best evidence of the strength dispersal, effort fragmentation, absence of clear goals, absence of integration of the society.

#### Evolution of cooperation

Robert Axelrod's book “The Evolution of Cooperation,” based on Game Theory, explains how cooperative behavior evolved and is maintained in humans (Axelrod and Hamilton, 1981). The study's goal was to develop a theory of what conditions are required for cooperation to emerge, particularly in situations where individuals can pursue their own interests without a central source of authority (e.g., societal rules or laws) to force cooperation through future consequences. Many of the benefits sought by living things are disproportionately available to cooperating groups (Axelrod and Hamilton, 1981, p. 1,391).

The origins and nature of our species' cooperative psychology and prosocial behavior have been a major scientific challenge since at least Darwin's time. However, with the rise of a highly interdisciplinary version of evolutionary psychology, progress on this question has accelerated, acknowledging our status as the “third chimp” while also recognizing that humans have evolved into a distinct cultural species. The issue of cooperation is concerned with how and why people make personal sacrifices to assist others (or avoid hurting them). Our species' incredible capacity for cooperation, in comparison to most other animals, has prompted researchers from a variety of fields to focus on understanding our “ultrasociality” (Campbell, 1983; Richerson and Boyd, 1998).

#### Concept of cooperation

Individuals must be able to collaborate and coordinate their actions when living in a group. In both space and time, cooperation in human societies extends far beyond kinship relationships. Humans' ability to cooperate with others, particularly strangers, challenges evolutionary theory because it appears to contradict the notion of “self-interest” as the primary motivator of behavior. Cooperation is defined as behavior that benefits others while not benefiting the individual directly. Although evolution theory considers cooperation with little or no cognition, human societies require advanced cognitive abilities. To establish and maintain complex cooperation, people must be able to recognize others' identities, states, and intentions, monitor their actual behavior, remember past interactions, calculate potential costs and benefits for long-term interactions, control one's behavior to receive a delayed reward, recognize deception, and other skills (Shkurko, 2021).

Those individuals work together for mutual benefit, which often comes at a cost to each participant at first, but the long-term benefits outweigh the costs. Cooperation is conceptually and empirically linked to altruism, which is defined as behavior in which one person foregoes a benefit to help another. Cooperation necessitates some level of altruism (at least at first). A person must first forego a benefit before cooperating. Altruism becomes cooperation when two or more people join forces to forego benefits to achieve a common goal that they believe is more valuable than the benefits foregone (Johnson et al., 2021).

#### Cooperation theory

The fundamental conflict that exists between what is good for the individual actor in the short run and what is good for the group in the long run is addressed by Cooperation Theory. The Prisoner's Dilemma is a particularly straightforward and compelling portrayal of this conflict. As a result, most Cooperation Theory research in a variety of fields now starts with the Prisoner's Dilemma. Other games, on the other hand, can be used to investigate aspects of the fundamental problem of cooperation that are not covered by the standard Prisoner's Dilemma.

Regardless of the theoretical details, game theory underpins almost all of Cooperation Theory. Game theory begins with actors who have choices. The outcome is jointly determined when the players make their choices. The payouts are determined by the outcome. Consider the two-person Prisoner's Dilemma with a single move. There are four possible outcomes if you cooperate or defect. The benefit of cooperation, R, outweighs the penalty of mutual defection, P. The defection payoff (T) is greater than the sucker payoff (S), creating a quandary. The Prisoner's Dilemma is defined by T>R>P>S. R > (S+T)/2 is commonly used to elevate cooperation above coordinated alternation of cooperation (Axelrod, 1984, 2000; Poundstone, 1992).

In a strategic context, game theory provides a very rich way of predicting outcomes. To specify a game, the players, the choices, the outcomes determined by the choices, and the player payoffs must all be specified. Just one more thing. It is necessary to predict how players will make decisions, or how they will select strategies in an iterated game. Traditionally, game theory predicted player behavior by assuming that players are rational, aware of the rationality of other players, and capable of infinite calculation. The assumption of rationalism (Axelrod, 2000).

##### Darwinian context

The theory of evolution that was proposed by Charles Darwin in “By Means of Natural Selection” (Hardin, 1968) is explicitly competitive and “survival of the fittest.” It is also explicitly Malthusian and “struggle for existence,” and even gladiatorial “survival of the fittest” and “nature, red in tooth and claw.” There is competition between species for shared resources, particularly between species that are similar in their needs and niches, as well as competition between individuals within the same species (Darwin, 1871). All of this comes down to one thing, and that is producing more offspring than all of your competitors and any and all of your predators.

Darwin's explanation of how the preferential survival of the smallest benefits can lead to advanced forms is not only one of the most powerful explanatory principles in biology but also one of the most important and influential in many other fields. This level of success has contributed to the widespread belief that life is fundamentally a conflict pitting each individual against the others, in which every individual is responsible for looking out for their own best interests and in which my success results in your failure.

##### Social darwinism

The evolution of cooperation is not an obscure technical issue of interest only to a small number of specialists because it reflects a larger issue at the intersection of political philosophy, ethics, and biology: the age-old question of individual interests vs. group interests. On the one hand, “Social Darwinists” (roughly, those who would use Darwinian evolution's “survival of the fittest” to justify the cutthroat competitiveness of laissez-faire capitalism); assert that the world is an inherently competitive “*dog eat dog*” jungle in which everyone must look out for himself (Bowler, 1984, p. 94–99, 269–70).

The Social Darwinists' point of view is based on Charles Darwin's interpretation of natural selection, which is explicitly competitive (survival of the fittest), Malthusian (struggle for existence), and even gladiatorial (red in tooth and claw) and pervaded by Darwin's and his disciples' Victorian laissez-faire ethos, such as T. H. Huxley and Herbert Spencer. Social Darwinists used what they read in the theory to support their social and economic beliefs, such as poverty being a natural condition and social reform being unnatural meddling (Bowler, 1984, p. 94–99).

#### Mechanisms for cooperation

##### Reciprocal altruism

Trivers (1971) proposed a plausible explanation for unrelated individuals helping one another: people help one another when they expect reciprocal behavior. There are two types of reciprocity that have been discussed so far. In its most basic form, direct reciprocity is a tit-for-tat cooperative strategy in which two individuals exchange cooperative acts and cease cooperating if one of them fails (Axelrod and Hamilton, 1981).

Recurring encounters between the same two individuals result in direct reciprocity (Trivers, 1971; Fudenberg and Maskin, 1986; Binmore and Samuelson, 1992; Sigmund, 2010). Because they interact so frequently, these people can use conditional strategies, in which their behavior is based on previous outcomes. Direct reciprocity allows cooperation to evolve if the likelihood of another interaction is high enough (Axelrod, 1984; Nowak and Sigmund, 1992, 1993).

##### Indirect reciprocity

In contrast, indirect reciprocity involves cooperation based on reputation (Nowak and Sigmund, 2005). Indirect reciprocation refers to situations in which someone other than the initial recipient provides a return favor. In this case, individuals assist those who are known to be helpful to others. To reap the benefits of cooperation, individuals must cultivate a cooperative reputation.

Indirect reciprocity occurs when there are repeated encounters within a population and third parties observe or learn about some of these encounters. Social media can spread information about such encounters, thereby affecting the reputations of the participants. Individuals can use conditional strategies to base their decisions on the reputation of the recipient (Nowak and Sigmund, 1998, 2005). What I do to you depends on what you did to me and others. Cooperation is costly, but it can increase your chances of receiving assistance from others by establishing a reputation as a helpful individual. The indirect reciprocity strategy consists of a social norm and an action rule (Brandt and Sigmund, 2006; Ohtsuki and Iwasa, 2006; Ohtsuki et al., 2009). The social norm describes how reputations of individuals are modified because of their interactions.

The action rule determines whether to cooperate based on the information available about the other person. Indirect reciprocity allows cooperation to develop when the probability of knowing someone's reputation is high enough. According to the theory of strong reciprocity, humans are predisposed to cooperate and punish non-cooperators, even at their own expense (Gintis, 2000). As a result, for humans to be inclined to cooperate, a trait of “cooperativeness” must have evolved and been selected (some more than others).

##### Spatial selection

Without the need for strategic complexity, spatial selection can favor cooperation (Nowak and May, 1992; Nowak et al., 2010). Behaviors do not need to be conditional on previous outcomes when populations are structured rather than randomly mixed. Cooperators can form clusters that win despite being surrounded by defectors because they interact with those around them. The basic idea is that clustering leads to assortment, which means that cooperators are more likely to interact with one another to get more gains. Population structure has an impact on the outcome of evolutionary processes in general, and some population structures can lead to the evolution of cooperation (Tarnita et al., 2009, 2011).

##### Multilevel selection

When there is competition between groups as well as competition between individuals in a group, this is called multilevel selection (Wilson, 1975; Boyd and Richerson, 1990; Sober and Wilson, 1998; Boyd et al., 2003; Traulsen and Nowak, 2006; Bowles, 2009; Bowles and Gintis, 2011). Defectors may win within their own groups, but cooperator groups do better than defector groups in the marketplace. In the end, this kind of process could lead to the selection of people who will work together. Darwin wrote in 1871 that there is no doubt that a tribe with many people who were always willing to help each other and give up themselves for the good of the group would win over other tribes. This would be an example of natural selection.

##### Kin selection

Kin selection describes how many species collaborate with one another (Hamilton, 1964). Even if it means sacrificing one's own survival, an allele that encourages helpful behavior toward kin could evolve and remain evolutionary stable. A person can pass on more of his or her genes to future generations by assisting relatives (who share one's genes). Individuals should be encouraged to collaborate with family members to improve their overall fitness. Cooperation among non-kin has been more difficult to explain because it is usually in the actor's best interest to act selfishly when interacting with non-kin.

Kin selection can be viewed as a mechanism for the evolution of cooperation if properly formulated. Kin selection occurs when there is conditional behavior based on kin recognition: when an individual recognizes their kin and acts accordingly. According to J.B.S. Haldane, “I will jump into the river to save two brothers or eight cousins” (Nowak and Highfield, 2011). Most of the current literature on kin selection, however, does not adhere to this simple definition based on kin recognition. In contrast, kin selection is associated with the notion of inclusive fitness (Hamilton, 1964).

Inclusive fitness is a mathematical approach to analyzing fitness effects. Personal fitness is assumed to be expressed as a sum of additive factors resulting from individual actions. Inclusive fitness is effective in certain situations, but it is based on strong assumptions that limit its applicability (Nowak et al., 2010). Proponents of inclusive fitness (Abbot et al., 2011) have contested this position, which is based on a careful mathematical analysis of evolution (Nowak et al., 2010, 2011). Only when the inherent limitations of inclusive fitness are widely acknowledged will a clear understanding of kin selection emerge. Meanwhile, keep in mind that no phenomenon in evolutionary biology necessitates a thorough fitness-based examination (Nowak et al., 2010).

#### Interactions between mechanisms

Each of these mechanisms is applicable to human cooperation. They were—and probably still are—all at work at some point during human evolution. Even though each mechanism has traditionally been studied separately, it is crucial to consider how they interact. When discussing the evolution of any prosocial behavior in humans, we cannot rule out direct and indirect reciprocity. Early human societies were small, so repetition and reputation were important (Rand and Nowak, 2013).

Even in the modern world, most of our significant interactions with friends and family are repeated. Considering their direct and indirect reciprocal interactions, spatial structure, group selection, and kin selection should all be considered. Combining mechanisms can produce unanticipated dynamics. Direct reciprocity and spatial structure may interact either synergistically or antagonistically, depending on the repetition and variety levels (van Veelen et al., 2012).

#### Types of cooperation

There are two types of cooperation, according to evolutionary theory. The first is largely unconditional and occurs between people who are related to one another, for example, kinship. Cooperation is based on pre-existing relationships with others in this case, and it is usually explained in terms of kin or group selection. Because it is dependent on specific social markers of a partner, this type of other-beneficial behavior is known as group-based cooperation. This means that an agent's decision to help others is influenced by their social affiliation or group membership with the agent. The second type of cooperation involves more strategic behavior and occurs between individuals who are not related to one another. In these situations, cooperation decisions are typically conditional, meaning they are based on expectations of mutuality and returns, which can be direct or indirect. Cooperation is a choice rather than an obligation, unlike the first type (Shkurko, 2021).

### Altruism and cooperation for social desirability

Cooperation and altruism have long been linked as interconnected prosocial behaviors, and the two are frequently studied together or thought to be equivalent constructs. It happens when two or more people collaborate to achieve a common goal (Tuomela, 1993). Cooperation entails both parties working toward a common goal, whereas altruistic behaviors are frequently one-sided and have no overt benefit to the party engaging in altruistic behavior.

Individuals engage in conditional cooperation when they are initially willing to take a risk and cooperate in a particular situation because they believe others will behave similarly in a subsequent interaction. This behavior could change if the partner or group members refuse to cooperate. This differs from traditional cooperation in that both parties are equally at risk in traditional cooperation, whereas in conditional cooperation, one party is willing to increase its risk in the hope that it will pay off in the future. Cooperation resembles reciprocal altruism because knowing the intentions of others is a key driver of cooperative behavior (Kocher et al., 2008).

### Gifted definition and identification

Increasing emphasis is being placed on meeting the educational, social, and psychological needs of gifted students. Multiple definitions exist for the term “gifted,” including those related to high intelligence quotient (IQ) and those that include multiple criteria that may not be measured by an IQ test. Diverse procedures for identifying gifted students and varying definitions of giftedness have been a persistent problem in the field, creating difficulties in identifying, placing, and providing appropriate services for gifted students (Bracken and Brown, 2006; Al-Hroub, 2010a,b, 2012, 2013, 2014, 2016). The problem stems mainly from using intelligence tests for assessing giftedness. Also, these intelligence tests used in the Arab world are imported from the West (primarily France and the United States) and translated into Arabic (Diab, 2006, p. 81). According to Al-Hroub (2013, 2014, 2016), these tests only provide an estimate of the students' intellectual ability, and IQ tests cannot be used as the sole criterion for determining giftedness. In addition to programmes designed for gifted students, therefore, it is necessary to provide more reliable and valid identification methods.

Students who perform well on intelligence tests are considered gifted. Those students who score 130 or higher in Stanford-Binet IQ scale are considered gifted students and are admitted to educational programmes for gifted students (Gallagher, 2015). Marland's initial definition emphasized categorical identification of gifted students who demonstrated achievement. Gifted and talented children are identified by professionally qualified individuals as being capable of high performance due to exceptional abilities. These are individuals who require differentiated educational programmes and/or services in addition to those provided by the regular school programme to contribute to society. Gifted students outperform their peers in any of the following areas, either individually or in combination: general cognitive ability, particular academic ability, thinking that is creative or productive, capability to lead, visual and performing arts, also known as psychomotor ability (p. 20–21).

The main concern of this study is the social-emotional aspect of giftedness, it adopted the behavioral and socio-emotional conceptualization of giftedness which help in easier and better identification of gifted students. Extensive empirical research indicates that gifted students are, on average, just as well-adjusted as their peers (Bain and Bell, 2004; Bracken and Brown, 2006; Cross et al., 2008; Mueller, 2009). Furthermore, a substantial body of research suggests that gifted students are disproportionately susceptible to a variety of social and emotional issues (Webb, 1994; Mendaglio and Peterson, 2006). Furthermore, recent research on gifted students' social-emotional functioning has been mixed at best. Positive traits include being less conforming to peer opinions and more independent (Gottfried and Gottfried, 1996), demonstrating better emotional adjustment (Oram et al., 1995), valuing cooperative and democratic forms of interaction (Lehman and Erdwins, 1981), demonstrating more leadership capabilities (Roeper, 1992), and being generally better psychologically adjusted (Howard-Hamilton and Franks, 1995; Nail and Evans, 1997).

#### The present study

The main interest of this study resides in taking the object of students enjoying special mental capabilities enabling them to reach a distinguished level in one or several fields, but it may be difficult for them to achieve that due to the failure in creating the conditions surrounding them and study their positive sides and prepare them (Alshakes, 2015). Review of studies in this area (Abu-Isac, 2001; Sweileh, 2001; Al-Ezah, 2002; Hussein, 2010; Elsheikh, 2016; Eskandarani, 2016; Mekki, 2016; Akkhateeb, 2017; Beheiry et al., 2017; Ali, 2020; Kasseem, 2020) reveals that altruism and cooperative behavior has been studied separately in relation with other psychological constructs, with a dearth studies dealing with cooperation in its relationship with altruism among a sample of gifted University students. Therefore, considering the researcher's sense of this phenomenon's appearance in the University students and based on what has been found of the great importance of these variables' influence on the disability, the idea of this research has emerged.

#### The study hypotheses

The study hypotheses are summed up as follows:

1. There is a positive relational significant correlation between cooperation and altruism with gifted University students.
2. There are significant differences between males and females in the cooperation and altruism of gifted University students.
3. There are significant differences between the junior and senior gifted students in both cooperation and altruism of gifted University students.

## Methods

### Participants

A total of 237 gifted University students from three Egyptian universities; Alexandria University, Sadat Academy for Management Sciences, and Suez University (in Egypt), and Kuwait University (State of Kuwait) were involved in this study. The three Egyptian universities are chosen to represent the far east and far west of Egypt, in addition to Kuwait University (the only government University in the State of Kuwait). Egyptian University gifted students represent 75.95% of participants whereas the students in Kuwait University participating in the study represent 24.05%. More than two thirds of the study samples are studying in scientific-oriented colleges 77.1%, students in the literary-oriented colleges represents only one third 22.9%. Participants were female (*N* = 144, 60.90%, average age 21.3 ± SD 2.6 years) and male (*N* = 93, 39.10%, average age 22.1 ± SD 2.7 years). The University gifted students live in their houses (*N* = 212, 89.71%) explains most respondents meanwhile the rest of the participants (*N* = 25, 10.29%) in this study live in the University dormitories (school hostels). Therefore, most of the respondents (77.1%) are from science-oriented colleges such as medicine and computer sciences, while the remaining 22.9% of the respondents were registered in literary-oriented colleges such as Education, Arts, and Management Sciences.

Gifted students participating in the study have been identified based on learning characteristics, creativity characteristics, motivation characteristics, leadership characteristics, artistic characteristics, musical characteristics, dramatics characteristics, communication characteristics (Precision), communication characteristics (expressiveness), planning characteristics, mathematics characteristics, reading characteristics, technology characteristics, and science characteristics (Renzulli et al., 2010).

### Measures

#### Generative altruism scale (GAlS)

In a German context, Büssing et al. (2013) developed the scale. It was originally made up of seven items that discussed specific helpful activities. Specific items are “*I help others even when there is no direct benefit*;” “*When I see individuals in need, I think about how to relieve their distress or meet their needs*;” and “*When I see individuals in need, I ask them how I can help*,” etc. All items were scored on a four-point scale measuring the intensity of the respective attitude or behavior (0—never; 1—sometimes; 2—often; 3—very often). Cronbach's Alpha was 0.93 in the original version of the scale, but it was 0.84 in this study.

#### The cooperative/competitive strategy scale (CCSS)

This scale was developed by Tang (1999). Tang created the CCSS (Cooperative/Competitive Strategy Scale; 1998). The survey includes 19 questions about cooperation, competition, and their relationship. There are eight questions about cooperation and 11 about competition. These 19 questions range from 1 for “always” to 7 for “never”, with one being “always.” On the right is a mean score for cooperation and competition. Cronbach's alpha of both cooperative and competitive behavior in the original scale were 0.73 and 0.74, respectively.

A study by Lu et al. (2012) found the CCSS to be valid, measuring both cooperative and competitive behavior (Cronbach's were 0.87 and 0.79, respectively). The results showed that the two-dimensional construct fit better than the one-factor model. In the current study, the authors argued that cooperativeness and competitiveness are distinct constructs and that the survey had significant predictive power for cooperative behavior in social dilemmas. Cronbach's alpha of the total survey was 0.84, and the Cronbach alpha for both cooperative and competitive behavior were 0.82 and 0.79, respectively.

## Results

Table 1 shows that Egyptian University gifted students represent 75.95% of participants whereas the students in Kuwait University participating in the study represent 24.05%. More than two thirds of the study samples are studying in scientific-oriented colleges 77.1%, students in the literary-oriented colleges represents only one third 22.9%. Participants were female (60.90%,) and male (39.10%). The University gifted students live in their houses (89.71%) explains most respondents meanwhile the rest of the participants (10.29%) in this study live in the University dormitories (school hostels).

**Table 1:** Descriptive statistics of the study sample.

	***N***	**%**	***N***	**%**
**University**				
Alex.	63	26.58%		
SAMS	43	18.14%		
Suez	74	31.22%		
KW	57	24.05%		
**Gender**				
Males			93	39.10%
Females			144	60.90%
**Residence**				
House	212	89.71%		
Dormitories	25	10.29%		
**Specialization**				
Science-oriented colleges			183	77.1%
Literary-oriented colleges			54	22.9%

The study hypothesized that there is a relationship between cooperation and altruism among gifted adolescents; there is a positive relational significant link between cooperation and altruism with the gifted University students, and Pearson correlation coefficients reveal that there is a significant correlation coefficient between cooperation and altruism. Results show that there is a significant positive relationship between the cooperation and altruism among males and females (*r*_males_ = 0.419, *p* < 0.01), (*r*_females_ = 0.401, *p* < 0.01), meanwhile a significant positive correlation coefficient was detected among the whole sample (*r*_total_ = 0.412, *p* < 0.01).

Table 2 shows Pearson correlation coefficients between cooperation and altruism of the means of males and females, revealing that there is a significant positive relation between cooperation and altruism among gifted adolescents.

**Table 2:** Pearson correlation coefficients between cooperation and altruism among gifted University students.

**Correlations**	**Males (*****N*** **= 93)**		**Females (*****N*** **= 144)**		**Total (*****n*** **= 237)**	
	**Altruism**	**Cooperation**	**Altruism**	**Cooperation**	**Altruism**	**Cooperation**
Cooperation	1	0.419**	1	0.401**	1	0.412**
Altruism	0.419**	1	0.401**	1	0.412**	1

** p < 0.01.

In addition, the study hypothesized that there are significant differences between males and females in both cooperation and altruism. *T*-test scores revealed that there are statistically significant differences between males and females in cooperation (*t*-value = 4.72, *p* < 0.01) and altruism (*t*-value = 5.92, *p* < 0.01).

Table 3 shows that there are statistically significant differences between males and females in favor of females in both cooperation and altruism. Also, the study hypothesized that there is a significant difference between high and low scores in both cooperation and altruism. *T*-test values revealed that there is a significant difference between high and low scores in both cooperation (*t*-value = 5.32, *p* < 0.01) and altruism (*t*-value = 5.67, *p* < 0.01).

**Table 3:** Mean scores, standard deviations, and *t*-test values of males and females in cooperation and altruism.

**Tests**	**Males (*****N*** **= 93)**		**Females (*****N*** **= 144)**		***T*-test**
	**M**	**SD**	**M**	**SD**	
Cooperation	47.56	4.35	61.88	6.77	4.72**
Altruism	32.72	3.13	39.88	8.84	5.92**

** Significant at the p < 0.01 level (2-tailed).

Table 4 reveals that there are statistically significant differences between senior and junior students' scores in both cooperation and altruism in favor of junior students.

**Table 4:** Mean scores, standard deviations, and *t*-test values of seniors and juniors on the cooperation and altruism scale.

**Tests**	**Senior students**		**Junior students**		***T*-test**
	**(*****N*** **= 108)**		**(*****N*** **= 129)**		
	**M**	**SD**	**M**	**SD**	
Cooperation	67.57	2.85	60.31	3.87	5.32**
Altruism	37.57	2.61	32.60	4.56	5.67**

** Significant at the 0.01 level (2-tailed).

## Discussion

The present findings provide further information on the relations between different dimensions of gifted adolescent cooperation and altruistic behavior. In addition, it identified the differences between males/females, and senior students/junior students in both cooperation and altruism. Two main findings result from our study on human altruistic and cooperative behaviors; (1) altruistic behaviors have been revealed to be closely linked to the cooperative attitudes of adolescents in University, (2) significant differences in both altruistic behaviors and cooperation in terms of males/females and senior/junior gifted students. Altruistic behavior is contingent on the altruism of others. However, people frequently have little or no knowledge of the altruistic reputation of others, for instance when the reputation was earned in a different social or economic context. Axelrod and Hamilton (1981) associate cooperation with altruism and competition restraint. Bernard (2014) examined the relationship between cooperative and competitive motivations and various personality disorders in a study.

When reputational information is incomplete, Ellers and van der Pool (2010) found that altruistic behavior is linked to cooperation considering the role of intrinsic expectation. When no information about altruistic reputation is available, an individual's expectations regarding the altruism of others are correlated with their generosity. Altruism and cooperation are two examples of prosocial behavior that can be conceptualized as taking different forms or being driven by different motivations (Carlo and Randall, 2002). The basis of altruistic motivation is an empathic concern. Altruism focuses on helping others without receiving any personal benefit (Batson et al., 1983; Eberly-Lewis and Coetzee, 2015). Cooperation is related to altruistic helping because it encourages prosocial behavior and places value on another person's wellbeing by emphasizing being helpful to people in need without waiting for any short or long-term benefits (Carlo and Randall, 2002; Batson et al., 2007).

The results of the study support the first hypothesis, which states that altruism and cooperation are positively related. Cooperation and altruism were found to have a significant correlation. This relationship can be explained by the fact that altruism is viewed as an adaptation and evolution of human sociality through cooperation (Pievani, 2011). In the Arabic context, there is a dearth of studies investigating the relationship between cooperative behavior and altruism. A most recent study in the Egyptian context (i.e., Ali, 2020) revealed that empathy is positively relates to gratitude as determinants of altruism among a sample of outstanding students.

Results of the current study showing a positive relationship between both altruism and cooperative behavior coincide with the study of Chakravarthy (2020) which indicated the role of personality on altruism and cooperation among young adults in India, it revealed that personality traits, altruism, and cooperation are significantly correlated. When examining generative altruism from the perspective of educational sciences in Muslim societies with diverse religious beliefs, it is necessary to focus on the relationships between students' generative altruism, religiosity/spirituality, and affective moral reasoning. The relationship between altruism and prosocial behavior (spirituality) was assured in more than one social and religious context; in the UK (Swank et al., 2012), Catholic-affiliated universities in the USA (Curry et al., 2009; Huber and Douglas, 2012), Muslim society in Turkey (Düzgüner, 2013; Sagir, 2020). In addition, altruism has been correlated with moral reasoning, especially in Muslim and middle eastern societies (Ersanli and Nurdan, 2015).

In comparison to their male counterparts, female gifted adolescents are more altruistic to others, display more cooperation in teamwork, and have enhanced positive attitudes toward working with others. They are also much more active team players. It appears that the normative judgments that adolescents acquire through socialization influence their altruistic behavior. There is also a link between adolescents' altruism and their internalization of hegemonic gender roles. Fan and Marini (2000) used longitudinal data to show that young women and men's gender-role attitudes are linked to their value orientations, which are derived from family socialization and gender-influenced. Furthermore, previous research has found that gender-specific socialization practices lead to differences in altruistic reasoning and behavior (Carlo et al., 1996). As a result, there's no reason to believe that adolescent perceptions of gender roles in society are unrelated to their gender beliefs.

Also, these results agree to some extent with results obtained through the study of Horn et al. (2021) which estimated unadjusted and adjusted gender gap in time preference, risk attitudes, altruism, trust, trustworthiness, cooperation, and competitiveness using data on high-school students. It was revealed that females are significantly more altruistic (both with classmates and schoolmates), and less competitive than males. Besides, Molina et al. (2013) revealed that there is a gender difference in cooperation in high school students in Spain that favors female students. Also, the study of Eswaran and Kotwal (2004) that dealt with a theory of gender differences in parental altruism, revealing that females outperformed their male counterparts.

In terms of the third hypothesis, the study's findings revealed that senior students, or older gifted students, are more altruistic and cooperative than junior or younger students. According to research conducted by Henrich et al. (2005) in 15 small societies, altruism is acquired gradually over the first two decades of life and then changes little thereafter. This emphasizes the importance of early socialization in the development of individual altruism: the incorporation of local cultural norms and values into individual preference functions during ontogeny (Gintis and Helbing, 2015).

Brocas et al. (2017) investigated the altruism and strategic giving in children and adolescents and the relationship between both altruism and strategic giving. We find that altruism increases with age in children and declines after adolescence, but it cannot explain the development of cooperation in the repeated game on its own. Older individuals reciprocate more and anticipate the potential benefits of initiating cooperative play more accurately. Overall, children under the age of seven are neither altruistic nor strategic, whereas college students cooperate strategically despite their relatively low altruism.

## Conclusion

This study's findings provide suggestive evidence of a relationship between altruism and cooperation. By strengthening or weakening them, counselors and clinicians would be better able to develop individualized treatment plans for their clients if they were aware of these factors in the counseling relationship. Intervention targeting increasing altruism and cooperative behaviors represent the standpoint for enhancing prosocial behaviors necessary for mental health, wellbeing, social efficacy, and adaptiveness. Helping others is associated with greater levels of cooperation, in addition to the benefits of receiving assistance and others. The implications for clinical interventions and future research are outlined, along with the connections between these findings and response shift theory.

The findings of these studies can be used to conduct further research with larger samples to prove beyond a reasonable doubt the predictability of altruism and cooperation in children, adolescents, and even adults. Working more specifically on the altruism levels and cooperative behavior of the client would promote behavioral activation and increase their wellbeing.

### Limitations

This research has limitations that could be addressed in future work. The results may have been influenced by the small sample size and the convenience of sampling. Self-report measures may influence participants to provide socially desirable responses. Comparing these variables across cultures would yield additional insights and contribute to the literature on altruism and cooperation. Utilizing a strength-based approach in a clinical setting could be facilitated by an understanding of the mechanism through which altruism influences changes in cooperative behavior. This would allow the counselors to develop a comprehensive understanding of the client's major psychological processes.

### Recommendations

The findings presented here have a wide range of implications for teachers, counselors, clinicians, parents, and administrators involved in the development of gifted children. Understanding social, behavioral, and affective needs of those individuals is essential if full development potential is to be maximized. Subsequent research is crucial to stand on socio-emotional and behavioral characteristics of gifted adolescents as a means to design interventions for supporting adaptive behaviors of this distinguished category of individuals.

## Data availability statement

The original contributions presented in the study are included in the article/supplementary material, further inquiries can be directed to the corresponding author.

## Ethics statement

All procedures followed were per the ethical standards of the responsible committee on human experimentation (Suez Canal University Committee of Scientific Research Ethics) and with the Helsinki Declaration of 1975, as revised in 2000. Informed consent was obtained from all students as well as their parents for being included in the study.

## Author contributions

All authors listed have made a substantial, direct, and intellectual contribution to the work and approved it for publication.

## Conflict of interest

The authors declare that the research was conducted in the absence of any commercial or financial relationships that could be construed as a potential conflict of interest.

## Publisher's note

All claims expressed in this article are solely those of the author and do not necessarily represent those of their affiliated organizations, or those of the publisher, the editors and the reviewers. Any product that may be evaluated in this article, or claim that may be made by its manufacturer, is not guaranteed or endorsed by the publisher.
